# Systematic review of self-management practices for vaginal irrigation among cervical cancer patients undergoing radiotherapy

**DOI:** 10.3389/fonc.2026.1807898

**Published:** 2026-06-03

**Authors:** Zhe Xu, Jiali Xu, Yi Gu, Yajie Ji, Ying Chen, Shanshan Zheng

**Affiliations:** 1 Department of Radiotherapy, The First Affiliated Hospital of Soochow University, Suzhou, China; 2 Department of Hematology, The First Affiliated Hospital of Soochow University, Suzhou, China

**Keywords:** cervical cancer, evidence-based nursing, radiotherapy, self-management, systematic review, vaginal irrigation

## Abstract

**Objective:**

To identify, evaluate, and summarize the best available evidence regarding the self - management behaviors of vaginal irrigation in patients undergoing radiotherapy for cervical cancer, in order to inform clinical practice.

**Methods:**

We systematically searched PubMed, Embase, Cochrane Library, Web of Science, and major Chinese databases (CNKI, Wanfang Data, SinoMed) for randomized controlled trials (RCTs) focusing on vaginal irrigation self - management in this patient population from the start until August 12, 2025. Although a search for high - level evidence such as guidelines and systematic reviews was planned, none met the inclusion criteria. Two researchers independently performed literature screening, quality assessment, and data extraction. The included evidence was graded, and recommendations were generated using the JBI (2014) evidence pre - grading and recommendation system.

**Results:**

A total of 23 RCTs were included. The best evidence was synthesized into 12 key recommendations across four domains: individualized management strategies, systematic health education, innovative supervision and promotion methods, and psychological and environmental support.

**Conclusion:**

Evidence from this review supports conditional recommendations (Grade B) for vaginal irrigation self - management, given the high risk of bias in the included RCTs. Healthcare professionals may consider these suggestions while integrating clinical expertise and patient preferences. Systematic, nurse - led management protocols tailored to local contexts may enhance adherence and reduce complications, pending higher - quality confirmatory research.

## 1 Introduction

Cervical cancer represents a significant global health challenge, ranking as the fourth most common cancer and the fourth leading cause of cancer death among women worldwide. According to recent WHO estimates, approximately 660,000 new cases and 350,000 deaths occurred globally in 2022, with nearly 90% of the mortality burden concentrated in low - and middle - income countries (1).

In China, the disease burden is particularly severe. The National Cancer Center reported approximately 150,000 new cases and 55,000 deaths in 2022, along with concerning trends toward younger onset (2).

Radiotherapy serves as a cornerstone in the management of locally advanced cervical cancer. Typically, it combines external beam radiation with brachytherapy as recommended by international guidelines (3, 4). While effective in improving survival, radiotherapy inevitably causes various complications. Radiation - induced vaginitis (45 - 60% incidence) and vaginal adhesions (16 - 30% incidence) are particularly prevalent issues that substantially impair the quality of life (5–7). Consequently, vaginal irrigation has been emphasized in clinical guidelines as an essential nursing intervention for removing necrotic tissue, preventing complications, and improving treatment outcomes (8, 9).

Despite established guidelines, adherence to vaginal irrigation protocols remains suboptimal in clinical practice. Studies indicate that only about 74 - 77% of patients achieve adequate knowledge mastery and compliance with irrigation procedures following conventional health education (10). The primary barriers include insufficient understanding of the procedure’s importance, technical skill deficits, psychological resistance, and inadequate ongoing professional support.

In response to these challenges, various interventions have been explored, including innovative health education models, enhanced continuity of care, and psychological support programs. Research by Zhang Xiaoyun et al. demonstrated the effectiveness of the Knowledge - Attitude - Practice model in improving irrigation adherence (18), while Zhao Haili et al. reported significant benefits from WeChat - based continuous care (15). Similarly, So KW et al. confirmed that structured health education programs could enhance self - care adherence and reduce complication risks (30). However, these valuable findings remain fragmented across individual studies without comprehensive synthesis, limiting their utility in guiding clinical practice.

To address this evidence - practice gap, this study employs systematic review methodology to identify, evaluate, and synthesize the best available evidence regarding vaginal irrigation self - management in cervical cancer patients undergoing radiotherapy. By integrating findings from multiple interventional studies, we aim to develop evidence - based recommendations that can inform the development of standardized management protocols, ultimately aiming to enhance patient adherence, reduce complications, and improve quality of life outcomes in this vulnerable patient population.

## 2 Materials and methods

### 2.1 Formulation of the evidence-based question

The PIPOST framework, developed by the Fudan University Centre for Evidence - Based Nursing, was utilized to define the clinical practice question. The components are as follows:

1. Population: Cervical cancer patients undergoing radiotherapy.
2. Intervention: Management strategies for vaginal irrigation self - management. Vaginal irrigation is defined as the therapeutic instillation of cleansing fluid into the vaginal canal to remove necrotic tissue, reduce radiation - induced inflammation, and prevent adhesions. Self - management is defined as the active process by which patients engage in health - promoting behaviors, monitor symptoms, and adhere to treatment regimens with professional support.
3. Professionals: Nurses.
4. Outcomes: Patient adherence to vaginal irrigation, quality of life, satisfaction, and related knowledge.
5. Setting: Wards in tertiary care hospitals and patients’ homes.
6. Type of evidence: Practice guidelines, clinical decisions, evidence summaries, expert consensus articles, systematic reviews, and randomized controlled trials (RCTs).

### 2.2 Search strategy

A comprehensive literature search was conducted based on the “6S” evidence pyramid model, aiming to systematically retrieve the highest level of available evidence from top - down. We conducted a comprehensive search across three tiers:

1. Guideline repositories: BMJ Best Practice, NICE guidance, National Guideline Clearinghouse (NGC);
2. International biomedical databases: PubMed, Embase, Cochrane Library, Web of Science;
3. Chinese databases: China National Knowledge Infrastructure (CNKI), Wanfang Data, SinoMed from inception to August 12, 2025.

**Figure 1:**
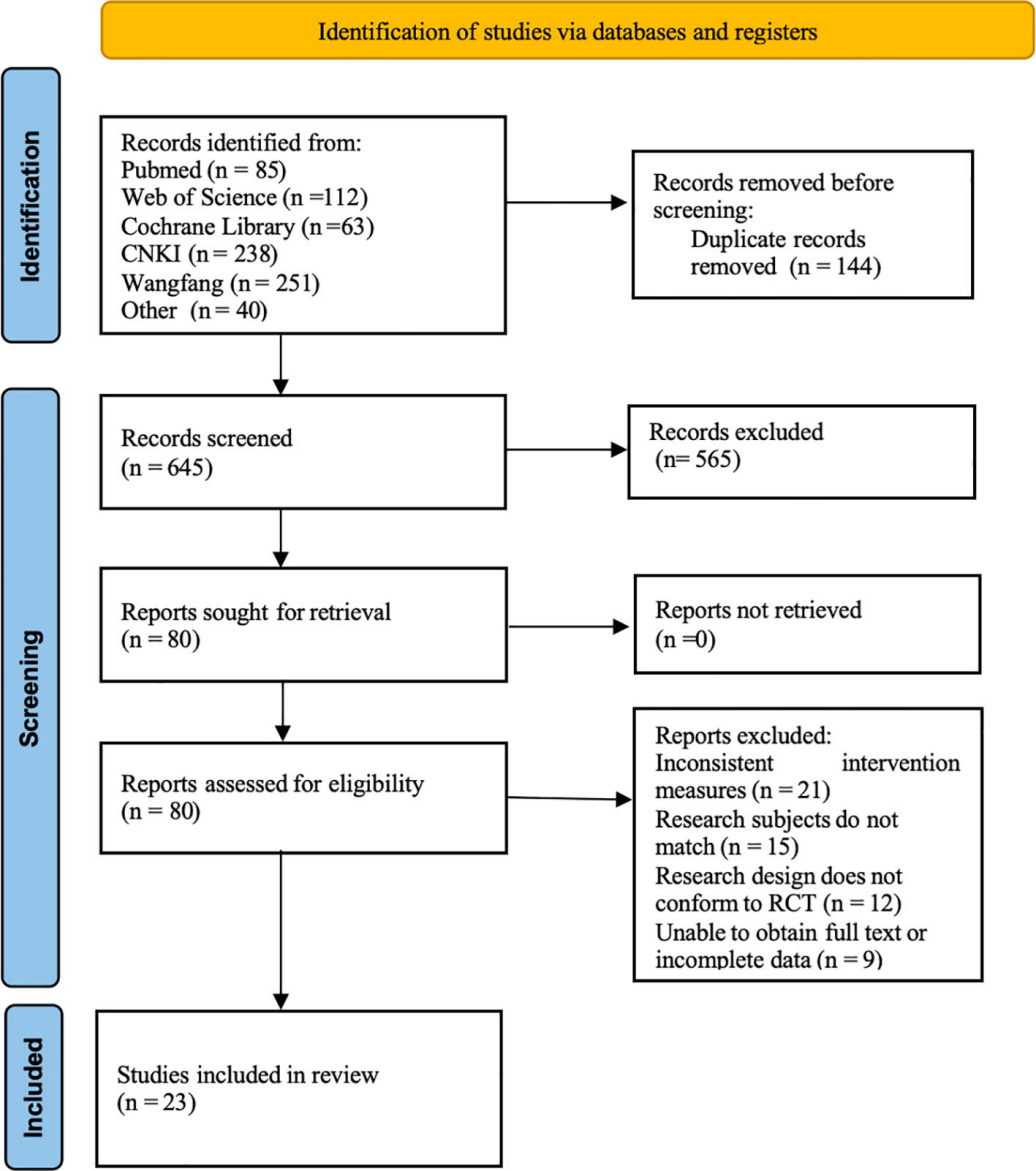
PRISMA 2020 flow diagram of study selection.

Complete search strategies for all databases are provided in **Supplementary Material 1**. The search encompassed clinical guidelines, expert consensuses, evidence summaries, systematic reviews, and RCTs related to vaginal irrigation in cervical cancer patients. This systematic review was conducted and reported in strict adherence to the Preferred Reporting Items for Systematic Reviews and Meta - Analyses (PRISMA) 2020 guidelines. The PRISMA 2020 flow diagram is presented in **Figure 1**, and the completed PRISMA 2020 checklist is available in **Supplementary Material 2**.

### 2.3 Literature inclusion and exclusion criteria

Inclusion criteria were:

1. Cervical cancer patients receiving radiotherapy;
2. Patients performing vaginal irrigation;
3. Studies involving interventions aimed at improving patients’ vaginal irrigation behavior;
4. Planned publication types included practice guidelines, clinical decisions, evidence summaries, expert consensuses, systematic reviews, and RCTs.

Exclusion criteria were:

1. Publications available only as abstracts, with full text inaccessible;
2. Publication types such as conference abstracts, conference papers, case reports, or basic science studies;
3. Superseded older versions of guidelines or their interpretations.

### 2.4 Literature screening and data extraction

Retrieved records were managed using Zotero software to remove duplicates. The screening process involved an initial review of titles and abstracts, followed by a full - text assessment of potentially eligible articles against the inclusion criteria. Two researchers independently performed all screening and data extraction steps. Any discrepancies were resolved through discussion or, if necessary, by consulting a third researcher.

### 2.5 Quality assessment

Two independent reviewers (Z.X. and J.X.) critically appraised the methodological quality of all included RCTs using the 2016 Joanna Briggs Institute (JBI) Critical Appraisal Checklist for Randomized Controlled Trials (13 items). Each item was rated as ‘Yes’, ‘No’, ‘Unclear’, or ‘Not applicable’ according to the JBI manual. Disagreements were resolved by discussion or consultation with a third reviewer (Y.G.). A detailed item - by - item assessment for each of the 23 studies is provided in **Supplementary Material 4**. Risk of bias was summarized by domain following JBI guidance. Overall risk of bias for each study was classified as low, moderate, or high based on the number and severity of ‘Unclear’ or ‘No’ ratings, with particular attention to key domains such as allocation concealment and blinding.

### 2.6 Evidence grading and synthesis

Given the complexity of behavioral interventions for self - management, we employed narrative evidence synthesis to integrate diverse intervention components targeting multiple adherence determinants. The 23 RCTs examined varied modalities including WeChat - based follow - up (n = 6), telephone support (n = 4), structured education programs (n = 5), innovative devices (n = 2), and multimodal combinations. These interventions differed in intensity, duration, and theoretical framework, making quantitative pooling inappropriate. The JBI FAME framework guided systematic categorization by intervention domain, preserving implementation details essential for clinical translation.

Evidence synthesis followed a three - stage process.

Stage 1: Data extraction. Two reviewers independently extracted intervention components, outcome measures, and effect directions (beneficial/harmful/no effect) from each study using a standardized form.

Stage 2: Content analysis. Extracted findings were categorized by intervention domain (individualized management, health education, supervision, psychological support). Within each domain, we assessed content consistency—findings were considered consistent if ≥70% of studies reporting the same outcome showed concordant effect directions.

Stage 3: Recommendation formulation. For consistent evidence, recommendations were drafted and graded using the JBI (2014) FAME (Feasibility, Appropriateness, Meaningfulness, Effectiveness) framework (31). Evidence levels ranged from 1 to 5, with recommendation strength classified as Grade A (strong) or B (weak).

Due to the pervasive high risk of bias across all 23 studies—specifically the absence of allocation concealment (selection bias) and blinding of participants, personnel, and outcome assessors (performance and detection bias)—the strength of all recommendations was downgraded to Grade B per the JBI FAME framework.

Management of Conflicting Evidence. When studies within the same domain reported discordant results (e.g., WeChat follow - up showing a positive effect in three studies but no effect in one), the following rules were applied:

1. Majority rule: If ≥70% of studies showed a consistent direction, the majority finding was adopted with notation of inconsistency;
2. Subgroup analysis: Heterogeneity was explored based on intervention intensity (e.g., frequency of follow - up) and population characteristics (e.g., age, disease stage);
3. Conservative interpretation: When no clear majority existed (<70% agreement), the recommendation was downgraded to Grade B or omitted.

Specific conflicts identified: Three studies (15, 16, 24) reported positive effects of WeChat follow - up on adherence, while one study (29) found no significant difference—attributed to a shorter intervention duration (4 weeks vs. 12 weeks); all were incorporated with notation of this moderator.

## 3 Results

### 3.1 Characteristics of included studies

The initial search identified 789 records (PubMed n = 142, Embase n = 35, Cochrane Library n = 78, Web of Science n = 121, CNKI n = 238, Wanfang Data n = 156, SinoMed n = 19). After removing 166 duplicates using Zotero software, 623 records remained for title and abstract screening.

Of these, 578 records were excluded as irrelevant, leaving 45 full - text articles to be assessed for eligibility. Twenty - two full - text articles were excluded with reasons: not RCT (n = 8), no vaginal irrigation intervention (n = 7), not cervical cancer patients undergoing radiotherapy (n = 4), conference abstracts with inaccessible full text (n = 3).

Ultimately, 23 RCTs were included in the narrative synthesis (10–30, 32, 33). No clinical guidelines, systematic reviews, or expert consensuses that fully met the predefined PIPOST criteria were identified.

Specifically:

1. Guidelines (e.g., NCCN Cervical Cancer Version 3.2025, Chinese Society for Radiation Oncology 2025 edition, National Health Commission 2022 edition, WHO Comprehensive Cervical Cancer Control) addressed broad radiotherapy management or general vaginal toxicities but contained no specific recommendations or protocols for vaginal irrigation self - management interventions.
2. Identified systematic reviews focused on radiation - induced vaginitis management, vaginal toxicities in general, or HPV - related douching risks, but none evaluated interventional strategies to improve patients’ self - management behaviors of vaginal irrigation.
3. Expert consensuses on intracavitary brachytherapy nursing discussed procedural irrigation during hospitalization but lacked home - based self - management components, follow - up support, or adherence outcomes.

No high - level evidence met all six PIPOST elements simultaneously. A full list with exact exclusion reasons and PIPOST mapping is provided in the new **Supplementary Material 5**. A PRISMA 2020 flow diagram detailing the study selection process is presented in **Figure 1**, and the list of excluded full - text studies with reasons is provided in **Supplementary Material 3**. The general characteristics of these included studies are presented in **Table 1**.

**Table 1:** Characteristics of included randomized controlled trials (n=23).

First author (year)	Source of publication	Primary focus of the study	Country
Huang SX (2024) (32)	VIP	Evaluation of an innovative vaginal irrigator in patients	China
Zhang J (2023) (33)	CNKI	Effect of continuous nursing guidance on compliance and quality of life	China
Sun LM (2023) (10)	Wanfang	Impact of health education on vaginal irrigation	China
Wang MM (2022) (11)	CNKI	Application of an innovative vaginal irrigator	China
Li XF (2021) (12)	CNKI	WeChat combined with precision nursing on patient rehabilitation	China
Long WY (2020) (13)	VIP	Effect of telephone follow-up on compliance	China
Lin SM (2020) (14)	CNKI	Comfort nursing and psychological intervention	China
Zhao HL (2019) (15)	CNKI	Continuous care via a WeChat follow-up platform	China
Li XL (2019) (16)	CNKI	Effect of WeChat follow-up on home compliance	China
Lu SZ (2019) (17)	CNKI	Impact of a distance care model on home compliance	China
Zhang XY (2018) (18)	CNKI	Effect of the KAP model on compliance	China
Zhang LF (2018) (19)	CNKI	Application of project management to improve irrigation rates	China
Ren QR (2018) (20)	Wanfang	Nursing effect of vaginal irrigation after radiotherapy	China
Ju XM (2017) (21)	CNKI	Application of humanistic nursing	China
Lu XY (2017) (22)	CNKI	Effect of continuous care on vaginal adhesion	China
Sun XY (2017) (23)	CNKI	Study on comfort nursing and psychological intervention	China
Shi XT (2016) (24)	Wanfang	Effect of WeChat follow-up on compliance and sexual life	China
Zhang Y (2016) (25)	CNKI	Application of a WeChat follow-up management platform	China
Liu DY (2016) (26)	CNKI	Observation on the effect of telephone follow-up	China
Wei WX (2015) (27)	Wanfang	Impact of telephone follow-up on vaginal irrigation	China
Chen Y (2014) (28)	CNKI	Systematic health education combined with a follow-up platform	China
Ji QJ (2017) (29)	CNKI	Continuous care via a network information platform	China
So WK (2005)	PubMed	Evaluation of an education program for women receiving internal radiation	China

KAP, Knowledge, Attitude, Practice; CNKI, China National Knowledge Infrastructure.

### 3.2 Results of quality assessment

Risk of bias assessment is summarized in **Table 2**, with detailed quality appraisal for individual studies provided in **Supplementary Material 4**. The methodological quality assessment revealed critical limitations across all 23 included RCTs. Most notably, no study reported allocation concealment (all rated ‘Unclear’), introducing a high risk of selection bias. Furthermore, blinding of participants and personnel was not implemented in any study due to the nature of behavioral interventions, resulting in a high risk of performance and detection bias. While baseline comparability and randomization were adequate, these pervasive methodological weaknesses substantially compromise the certainty of the evidence. Consequently, the overall certainty of the evidence should be regarded as low rather than moderate, and all recommendations derived from this evidence base should be interpreted as conditional rather than definitive.

**Table 2:** Summary of risk of bias assessment across 23 included RCTs using the JBI Critical Appraisal Checklist (n=23).

Quality domain	Finding
Randomization sequence generation	Reported in all studies (100%)
Allocation concealment	Not reported in any study (0%; high risk)
Blinding of participants and personnel	Not feasible in any study (0%; high risk)
Blinding of outcome assessors	Not reported in any study (0%; high risk)
Incomplete outcome data	Adequately addressed in 18 studies (78%)
Selective reporting	Low concern in 20 studies (87%)
Overall risk of bias	High (all 23 studies)

High risk of bias was identified in the following domains across all studies: allocation concealment (0% reported = high risk of selection bias) and blinding of participants, therapists, and outcome assessors (0% feasible = high risk of performance and detection bias). These are the two most common sources of bias in behavioral intervention trials. Full per-study ratings for all 13 items are presented in **Supplementary Material 4**.

### 3.3 Summary of evidence

Following a comprehensive synthesis of the extracted findings, the evidence was categorized into 12 distinct recommendations across four primary domains: Individualized Management Strategies, Systematic Health Education, Innovative Supervision and Promotion Methods, and Psychological and Environmental Support. The complete evidence summary, including the specific recommendations, their evidence levels, and strength of recommendation, is detailed in **Table 3**.

**Table 3:** Summary of best evidence for self-management of vaginal irrigation (n=23 RCTs).

No.	Domain	Recommendation	Level	Grade	Brief Rationale (Support)
1	Individualized Management	Develop personalized management plans based on disease status, cultural background, and support system	1b	B	tailored plans significantly improved adherence and reduced complications by addressing individual barriers (10, 14, 19, 21).
2		Conduct dynamic evaluations using record cards, reminders, and patient feedback	1b	B	Regular feedback and reminders enhanced ongoing compliance and early detection of issues in multiple studies (12, 15, 18, 24).
3	Health Education	Utilize diverse educational formats (manuals, videos, lectures, demonstrations, remote education)	1b	B	Multimodal education formats improved knowledge mastery and skill acquisition compared to standard methods (13, 15, 18, 30).
4		Provide individualized skills training with one-on-one coaching and on-site assessment	1b	B	One-on-one coaching and demonstrations led to higher procedural accuracy and self-efficacy (14, 19, 21, 30).
5		Provide clear operational guidance†	1b	B	Structured guidance on parameters (e.g., temperature, depth) reduced errors and improved standardization (11, 19, 22, 32).
6	Supervision & Promotion	Establish collaborative supervision network (nurses, patients, family, electronic platforms)	1b	B	Multi-component networks (e.g., nurse-physician-family) created closed-loop support and markedly boosted adherence (15, 19, 30).
7		Facilitate peer support networks for mutual supervision and experience sharing	1b	B	Peer sharing reduced isolation and reinforced positive behaviors in behavioral interventions (18, 25).
8		Combine WeChat platform support with telephone follow-ups	1b	B	Hybrid digital + telephone follow-up overcame barriers to home adherence, with consistent positive effects (12, 15, 16, 24).
9		Consider innovative devices (e.g., catheters for deep irrigation)	1b	B	Novel devices improved irrigation depth and comfort, leading to better compliance in targeted studies (11, 32).
10	Psychological & Environmental	Integrate humanistic care throughout treatment	1b	B	Humanistic approaches alleviated psychological resistance and improved overall satisfaction (14, 17, 20, 23).
11		Provide professional psychological counseling with anxiety/depression scale assessment	1b	B	Targeted counseling reduced anxiety/depression and supported adherence in radiotherapy patients (14, 17, 20).
12		Optimize treatment environment with emphasis on privacy protection	1b	B	Privacy measures decreased embarrassment and increased willingness to perform self-management (17, 20, 23).

Grade adjustment rationale: Although evidence level is 1b (RCTs), all recommendations are downgraded to Grade B (weak/conditional) due to high risk of bias (no allocation concealment, no blinding) across all included studies. Grade A: Strong recommendation; Grade B: Weak recommendation.

*Operational parameters: Fluid temperature 41–43 °C, depth 6–8cm, duration ~15min reported in studies (11, 19, 22, 32). Body temperature ≤37.5 °C as safety threshold was mentioned in 4 studies but represents extrapolation from general gynecological procedural guidelines rather than vaginal irrigation-specific evidence. None of the included RCTs experimentally validated optimal safety parameters. These parameters require individualized adaptation; rigid adherence is discouraged.

For example, Evidence Item 6 (collaborative supervision network) synthesizes findings from Zhang et al. (19) (project management model), Zhao et al. (15) (WeChat platform), and So et al. (30) (structured education), all of which demonstrate improved adherence through multi - component interventions.

Operational parameters (Item 5) warrant specific caution. While 4 studies (11, 19, 22, 32) reported fluid temperature ranges of 41 - 43 °C, none experimentally validated these parameters or the body temperature safety threshold (≤37.5 °C). The 37.5 °C cutoff, mentioned in 4 studies as an exclusion criterion, reflects general infection control principles extrapolated from broader gynecological procedural guidelines (e.g., hysteroscopy) rather than direct evidence from vaginal irrigation research.

Consequently, these parameters should serve as context - dependent practical guidance only, requiring individualized adjustment based on patient anatomy, comfort tolerance, and clinical circumstances rather than rigid adherence.

## 4 Discussion

This systematic review synthesized evidence from 23 RCTs with a high risk of bias, offering 12 conditional recommendations (Grade B) across four domains for vaginal irrigation self - management in cervical cancer patients undergoing radiotherapy. Preliminary evidence suggests that a structured, nurse - coordinated model may enhance irrigation adherence.

As demonstrated by Zhang et al. (19), a multidisciplinary supervision team involving nurses, physicians, patients, and families significantly enhances irrigation adherence and standardization, thereby reducing complication rates. Within this framework, nurses execute, guide, and monitor the regimen while facilitating communication; physicians provide specialized input; and patients and families engage in decision - making and feedback, establishing a closed - loop management system.

To ensure the continuity and effectiveness of self - management plans, healthcare institutions are encouraged to actively develop such collaborative mechanisms with clearly defined roles.

Furthermore, the integration of dynamic knowledge dissemination and sustained remote supervision may be important for improving long - term home - based care. Multiple studies (19, 22–24) confirm that nurse - led supervision, utilizing platforms like WeChat for educational materials, instructional videos, and personalized reminders, effectively transcends spatial and temporal barriers. This approach markedly improves the standardization and adherence to vaginal irrigation at home, aligning with the findings of So et al. (30).

This evolution expands the scope of continuous nursing from mere “information delivery” to comprehensive “behavioral supervision and support.” Future initiatives should address current limitations, such as monotonous supervision channels and homogenized content, by developing more personalized and interactive remote monitoring solutions.

Additionally, a major limitation of this systematic review is that the evidence base is predominantly China-centric. Of the 23 included RCTs, 22 were conducted in mainland China and only one was carried out in a Chinese-speaking population in Hong Kong. This heavy reliance on studies from a single cultural and healthcare context inevitably limits the direct generalizability of the findings to other regions of the world, including Western countries, Southeast Asian nations, African countries, Latin America, and other low- and middle-income settings. Factors such as cultural norms regarding bodily modesty, sexuality, and gender roles, differences in healthcare infrastructure and resource availability, varying patient expectations and health literacy levels, as well as distinct family structures and decision-making patterns, may differ substantially from those observed in the Chinese studies. Consequently, caution should be exercised when attempting to apply these recommendations directly outside of East Asian contexts.

Nevertheless, we argue that the core behavioral and nursing principles synthesized in this review possess considerable universal value. Elements such as systematic and multimodal health education, individualized skills training with one-on-one coaching, nurse-led remote supervision and follow-up, active family involvement, peer support networks, integration of psychological counseling, and strong emphasis on privacy protection address fundamental determinants of self-management behavior that are relevant across diverse populations. Although the specific technological modalities — particularly the widespread use of WeChat-based platforms for continuous care and reminders — are highly context-specific and may not be directly transferable, the underlying nurse-coordinated, multi-modal, closed-loop supervision model demonstrates strong conceptual parallels with successful international self-management programs for cancer-related toxicities and other chronic illnesses.

For successful cross-cultural adaptation in non-Chinese settings, several important considerations must be addressed. Clinicians and policymakers should substitute WeChat with locally appropriate and culturally acceptable digital or non-digital platforms that can deliver comparable functions, such as WhatsApp, Telegram, LINE, hospital patient portals, SMS messaging systems, or even traditional telephone follow-up combined with printed materials. Moreover, educational content, instructional videos, and psychological support strategies need to be carefully tailored to local cultural norms, especially concerning discussions of vaginal health, sexual function, and self-care practices that may carry different levels of stigma or embarrassment across societies. In more individualistic cultures, greater emphasis may need to be placed on patient autonomy and self-efficacy, whereas in collectivist societies, family involvement may remain central to the supervision network. Local contextual assessments, including qualitative explorations of patient and healthcare provider perspectives, barriers related to privacy and cultural taboos, and evaluations of feasibility and acceptability, are strongly recommended before large-scale implementation. Ultimately, rigorous adaptation studies and pragmatic international randomized controlled trials conducted in diverse healthcare systems are urgently needed to confirm the transferability of these findings, refine context-specific implementation strategies, and evaluate the effectiveness, cost-effectiveness, and long-term impact of adapted vaginal irrigation self-management protocols in global cervical cancer care.

A critical challenge identified in this review pertains to the operational parameters for vaginal irrigation (Evidence Item 5). The commonly cited values for fluid temperature, insertion depth, and duration, along with the body temperature safety threshold, have never been experimentally validated in any of the 23 included randomized controlled trials (RCTs). These parameters are practice-based rather than evidence-based, as they are derived solely from clinical experience and expert consensus and extrapolated from unrelated gynecological procedures. Consequently, they should be treated only as flexible reference points, applied with strict individualization according to patient anatomy, comfort tolerance, and real-time monitoring.

This evidence gap underscores the urgent need for high-quality optimization RCTs that directly compare different parameter combinations to establish safe, effective, and standardized evidence-based protocols.

The translational application of these findings may face challenges, including variability in institutional resources, nurses’ evidence-based practice competencies, and patient acceptance. Healthcare administrators should, therefore, conduct thorough contextual assessments prior to implementation, prioritize clinical issues, and devise systematic plans encompassing staff training, process redesign, and quality monitoring. Concurrently, local application studies based on this evidence are encouraged to validate its effectiveness and explore optimal models suitable for diverse healthcare settings.

It is imperative to recognize the methodological limitations of the included RCTs, which fundamentally affect the strength of our conclusions. The most notable concern is the widespread lack of reported allocation concealment, introducing a high risk of selection bias. Additionally, the nature of the interventions precluded blinding of participants and personnel, potentially contributing to performance and detection bias. Although the evidence originated from Level 1b (RCTs), the uniform high risk of bias resulted in Grade B (conditional) recommendations for all 12 items. These should be regarded as practice suggestions rather than definitive directives.

Narrative synthesis enabled comprehensive integration of multifaceted behavioral interventions that would be inappropriate for meta-analytic pooling due to differing mechanistic targets. This approach preserved critical implementation details—such as platform features, training protocols, and supervision intensity—that facilitate real-world application. While quantitative effect estimates were not generated, the structured recommendations provide actionable guidance adaptable to diverse clinical contexts. In practice, these should be regarded as “conditional recommendations” or “practice suggestions.” Clinicians must apply these findings judiciously, integrating professional expertise and individual patient circumstances.

Future research must prioritize methodological rigor, particularly the explicit implementation and reporting of allocation concealment, to generate more reliable evidence.

This review has several important limitations.

First, the methodological quality of included RCTs fundamentally constrains our conclusions. All 23 studies lacked allocation concealment (high risk of selection bias) and blinding (high risk of performance and detection bias). Consequently, all recommendations in **Table 3** should be interpreted as conditional (Grade B) rather than definitive, requiring integration with clinical expertise and patient preferences.

Second, most participants received combined EBRT plus brachytherapy, but the lack of subgroup analyses limits our ability to provide modality-specific recommendations for vaginal irrigation self-management. Third, the evidence base is predominantly China-centric, with 22 of the 23 included RCTs conducted in mainland China and only one in Hong Kong. This geographic concentration may limit the generalizability of the findings to other regions with different cultural norms, healthcare infrastructure, and patient expectations. Fourth, the restriction to Chinese and English publications may introduce language bias. Fifth, the evidence base consists solely of RCTs; exclusion of grey literature means practical contextual details were not incorporated.

Sixth, the search date (August 12, 2025) means recently published studies might not have been indexed.

These limitations highlight the need for future high-quality RCTs with explicit allocation concealment, blinded outcome assessment, and stratification by radiotherapy modality (EBRT versus brachytherapy).

A significant finding of this review is the exclusive reliance on RCTs because of the absence of eligible high - level evidence, such as guidelines or systematic reviews. This indicates a notable gap in the current evidence landscape for this specific practice area.

Future efforts should, therefore, focus on two parallel tracks: the development of detailed, operational clinical practice guidelines and the execution of comprehensive systematic reviews and meta - analyses of existing RCTs to generate higher - level syntheses.

## 5 Conclusion

This systematic review synthesizes evidence from 23 RCTs on self - management of vaginal irrigation in cervical cancer patients undergoing radiotherapy, presenting 12 conditional recommendations (Grade B) across four domains. Clinicians should apply these findings with caution, integrating clinical expertise and patient preferences. Healthcare professionals in non - Chinese contexts should adapt these suggestions to local platforms and sociocultural circumstances while retaining the core elements of nurse - led supervision. Future high - quality RCTs with allocation concealment are required to strengthen this evidence base. 
