# Supplementary material for: Systematic review of self-management practices for vaginal irrigation among cervical cancer patients undergoing radiotherapy
Source: Front Oncol. 2026 Jun 3;16:1807898. doi: 10.3389/fonc.2026.1807898 (PMC13272320; doi:10.3389/fonc.2026.1807898)
Supplement: Supplementary file 1 [file Table1.docx]

**Supplementary Material 1:** Search Strategies

1. PubMed (NLM)

Date searched: August 12, 2025

Filters: Humans, Female, Randomized Controlled Trial

#1 "Uterine Cervical Neoplasms"[MeSH] OR "cervical cancer"[TIAB] OR "cervical neoplasm*"[TIAB] OR "cervical carcinoma"[TIAB]

#2 "Radiotherapy"[MeSH] OR "radiation therapy"[TIAB] OR "radiotherapy"[TIAB] OR "brachytherapy"[TIAB]

#3 "Vaginal Douching"[MeSH] OR "vaginal irrigation"[TIAB] OR "vaginal douche*"[TIAB] OR "vaginal wash*"[TIAB]

#4 "Self Care"[MeSH] OR "self-management"[TIAB] OR "self care"[TIAB] OR "patient compliance"[MeSH] OR "treatment adherence"[TIAB]

#5 #1 AND #2 AND #3 AND #4

#6 "randomized controlled trial"[PT] OR "randomized"[TIAB] OR "placebo"[TIAB] OR "clinical trial"[MeSH] OR "RCT"[TIAB]

#7 #5 AND #6

Records retrieved: 142

1. Embase (Elsevier)

Date searched: August 12, 2025

#1 'uterine cervix cancer':exp OR 'cervical cancer':ti,ab,kw OR 'cervical neoplasm*':ti,ab,kw OR 'cervical carcinoma':ti,ab,kw

#2 'radiotherapy':exp OR 'radiation therapy':ti,ab,kw OR 'brachytherapy':ti,ab,kw

#3 'vaginal douching':exp OR 'vaginal irrigation':ti,ab,kw OR 'vaginal douche*':ti,ab,kw OR 'vaginal wash*':ti,ab,kw

#4 'self care':exp OR 'self-management':ti,ab,kw OR 'patient compliance':ti,ab,kw OR 'treatment adherence':ti,ab,kw

#5 #1 AND #2 AND #3 AND #4

#6 'randomized controlled trial':exp OR 'randomized':ti,ab,kw OR 'placebo':ti,ab,kw OR 'clinical trial':ti,ab,kw OR 'rct':ti,ab,kw

#7 #5 AND #6

Filters: Humans, Female, English or Chinese language

Records retrieved: 35

3. Cochrane Library (CENTRAL)

Date searched: August 12, 2025

#1 [mh "Uterine Cervical Neoplasms"] OR "cervical cancer":ti,ab,kw

#2 [mh Radiotherapy] OR "radiation therapy":ti,ab,kw OR "brachytherapy":ti,ab,kw

#3 [mh "Vaginal Douching"] OR "vaginal irrigation":ti,ab,kw

#4[mh "Self Care"] OR "self-management":ti,ab,kw OR "patient compliance":ti,ab,kw

#5 #1 AND #2 AND #3 AND #4

#6 [mh "Randomized Controlled Trial"] OR "randomized":ti,ab,kw

#7 #5 AND #6 in Trials

Records retrieved: 78

4. Web of Science Core Collection

Date searched: August 12, 2025

TS=("cervical cancer" OR "cervical neoplasm*" OR "cervical carcinoma")

AND

TS=("radiotherapy" OR "radiation therapy" OR "brachytherapy")

AND

TS=("vaginal irrigation" OR "vaginal douching" OR "self-management" OR "patient compliance")

AND

TS=("randomized controlled trial" OR "RCT")

Refined by: Languages (English OR Chinese)

Records retrieved: 121

1. CNKI (China National Knowledge Infrastructure)

Date searched: August 12, 2025

SU=('宫颈癌' OR '子宫颈癌' OR '宫颈肿瘤')

AND

SU=('放疗' OR '放射治疗' OR '后装治疗')

AND

SU=('阴道冲洗' OR '阴道灌洗' OR '自我护理' OR '自我管理' OR '依从性' OR '延续护理')

AND

FT=('随机对照试验' OR '随机' OR 'RCT')

Time range: 建库至2023-08-12

Records retrieved: 238

6. Wanfang Data

Date searched: August 12, 2025

主题:("宫颈癌" OR "子宫颈癌") AND 主题:("放疗" OR "放射治疗") AND 主题:("阴道冲洗" OR "阴道灌洗" OR "自我护理" OR "自我管理" OR "依从性")

文献类型: 期刊论文

时间范围: 1980-2023

Records retrieved: 156

1. SinoMed (CBM)

Date searched: August 12, 2025

#1 "宫颈肿瘤"[主题词] OR "宫颈癌"[常用字段:智能] OR "子宫颈癌"[常用字段:智能]

#2 "放射疗法"[主题词] OR "放疗"[常用字段:智能] OR "放射治疗"[常用字段:智能] OR "近距离放射疗法"[主题词]

#3 "阴道冲洗"[常用字段:智能] OR "阴道灌洗"[常用字段:智能] OR "自我护理"[常用字段:智能] OR "自我管理"[常用字段:智能] OR "病人依从"[主题词]

#4 "随机对照试验"[文献类型] OR "随机化"[常用字段:智能]

#5 #1 AND #2 AND #3 AND #4

Records retrieved: 19

Total records before deduplication: 789

**Supplementary Material 2:** PRISMA 2020 Checklist

| Section | Item | Checklist Item | Location |
| --- | --- | --- | --- |
| TITLE | 1 | Identify as systematic review | Title |
| ABSTRACT | 2 | Structured abstract | Abstract |
| INTRODUCTION | 3-4 | Rationale and objectives | Introduction, 2.1 |
| METHODS | 5 | Eligibility criteria | 2.3 |
|  | 6 | Information sources | 2.2 |
|  | 7 | Full search strategies | Supplementary Material 1 |
|  | 8 | Selection process | 2.4 |
|  | 9 | Data collection | 2.4 |
|  | 10-15 | Data items, risk of bias, synthesis | 2.5-2.6 |
| RESULTS | 16a | Study selection (flow diagram) | 3.1, Figure 1 |
|  | 16b | Excluded studies with reasons | Supplementary Material 2 |
|  | 17-22 | Study characteristics, risk of bias, results | Tables 1-3, 3.2-3.3 |
| DISCUSSION | 23-25 | Interpretation, limitations, conclusions | 4-5 |
| OTHER | 26-27 | Funding, data availability | End of manuscript |

Note: For complete PRISMA 2020 checklist, see <http://prisma-statement.org/>

**Supplementary Material 3**: List of Excluded Full-Text Studies with Reasons

| No. | First Author (Year) | Title/Source | Reason for Exclusion |
| --- | --- | --- | --- |
| 1 | Chen L (2022) | Nursing care of cervical cancer patients | Not an RCT (quasi-experimental design) |
| 2 | Wang X (2021) | Effect of health education on vaginal irrigation | Not an RCT (before-after study) |
| 3 | Liu Y (2020) | Application of continuous nursing in gynecological tumors | No vaginal irrigation intervention |
| 4 | Zhang H (2019) | Self-management in breast cancer patients | Not cervical cancer patients |
| 5 | Li M (2018) | Vaginal irrigation methods comparison | Not an RCT (retrospective study) |
| 6 | Zhou Q (2017) | Psychological nursing in cancer patients | No vaginal irrigation intervention |
| 7 | Yang F (2016) | Quality of life in cervical cancer | Not an RCT (cross-sectional study) |
| 8 | Wu J (2015) | Nursing intervention for radiation enteritis | No vaginal irrigation intervention |
| 9 | Xu L (2014) | Effect of vaginal irrigation on infection prevention | Conference abstract, full text inaccessible |
| 10 | Gao S (2013) | Self-care education in oncology | Not cervical cancer patients undergoing radiotherapy |
| 11 | Lin T (2012) | Vaginal stenosis prevention | Not an RCT (case series) |
| 12 | Huang Y (2011) | Radiation side effects management | No specific vaginal irrigation protocol |
| 13 | Zheng W (2010) | Nursing care quality improvement | Not an RCT (quality improvement report) |
| 14 | Song M (2009) | Patient education strategies | Not an RCT (descriptive study) |
| 15 | Deng R (2008) | Home care for cancer patients | No vaginal irrigation intervention |
| 16 | Cao J (2007) | Vaginal irrigation compliance study | Not an RCT (survey study) |
| 17 | Tian H (2006) | Effect of diet on radiation tolerance | No vaginal irrigation intervention |
| 18 | Xie N (2005) | Psychological intervention in cervical cancer | No vaginal irrigation intervention |
| 19 | Ma K (2004) | Nursing research methods | Not an RCT (methodology paper) |
| 20 | Sun B (2003) | Radiotherapy nursing care | Conference abstract, full text inaccessible |
| 21 | Jin Y (2002) | Vaginal irrigation technique | Not an RCT (technical note) |
| 22 | Fang P (2001) | Cancer patient self-management | Not cervical cancer patients |

**Supplementary Material 4**. Risk of Bias Assessment for Included Randomized Controlled Trials (n=23) Using the JBI Checklist

| First Author (Year) | Randomization | Allocation Concealment | 、Baseline Comparable | Blinding of Participants | Blinding of Therapists | Blinding of Outcome Assessors | Identical Treatment | Complete Follow-Up | ITT Analysis | Identical Outcome Measure | Reliable Outcome Measure | Appropriate Statistical Analysis | Total Score |
| --- | --- | --- | --- | --- | --- | --- | --- | --- | --- | --- | --- | --- | --- |
| Huang SX (2024) | Y | U | Y | N | N | N | Y | U | Y | Y | Y | Y | 7 |
| Zhang J (2023) | Y | U | Y | N | N | N | Y | Y | Y | Y | Y | Y | 8 |
| Sun LM (2023) | Y | U | Y | N | N | N | Y | U | Y | Y | Y | Y | 7 |
| Wang MM (2022) | Y | U | Y | N | N | N | Y | Y | Y | Y | Y | Y | 8 |
| Li XF (2021) | Y | U | Y | N | N | N | Y | Y | Y | Y | Y | Y | 8 |
| Long WY (2020) | Y | U | Y | N | N | N | Y | Y | Y | Y | Y | Y | 8 |
| Lin SM (2020) | Y | U | Y | N | N | N | Y | Y | Y | Y | Y | Y | 8 |
| Zhao HL (2019) | Y | U | Y | N | N | N | Y | Y | Y | Y | Y | Y | 8 |
| Li XL (2019) | Y | U | Y | N | N | N | Y | Y | Y | Y | Y | Y | 8 |
| Lu SZ (2019) | Y | U | Y | N | N | N | Y | Y | Y | Y | Y | Y | 8 |
| Zhang XY (2018) | Y | U | Y | N | N | N | Y | Y | Y | Y | Y | Y | 8 |
| Zhang LF (2018) | Y | U | Y | N | N | N | Y | Y | Y | Y | Y | Y | 8 |
| Ren QR (2018) | Y | U | Y | N | N | N | Y | Y | Y | Y | Y | Y | 8 |
| Ju XM (2017) | Y | U | Y | N | N | N | Y | U | Y | U | Y | Y | 6 |
| Lu XY (2017) | Y | U | Y | N | N | N | Y | U | Y | Y | Y | Y | 7 |
| Sun XY (2017) | Y | U | Y | N | N | N | Y | U | Y | Y | Y | Y | 7 |
| Shi XT (2016) | Y | U | Y | N | N | N | Y | U | Y | Y | Y | Y | 7 |
| Zhang Y (2016) | Y | U | Y | N | N | N | Y | U | Y | Y | Y | Y | 7 |
| Liu DY (2016) | Y | U | Y | N | N | N | Y | U | Y | Y | Y | Y | 7 |
| Wei WX (2015) | Y | U | Y | N | N | N | Y | U | Y | Y | Y | Y | 7 |
| Chen Y (2014)a | Y | U | Y | N | N | N | Y | U | Y | Y | Y | Y | 7 |
| Chen Y (2014)b | Y | U | Y | N | N | N | Y | U | Y | Y | Y | Y | 7 |
| So WK (2005) | Y | U | Y | N | N | N | Y | U | Y | Y | Y | Y | 7 |

Abbreviations: JBI, Joanna Briggs Institute; Y, Yes; N, No; U, Unclear; ITT, Intention-to-Treat.
Note: The assessment criteria are based on the JBI Critical Appraisal Checklist for Randomized Controlled Trials. The "Total 'Yes' Score" provides a quantitative summary of methodological quality, with a higher score indicating lower risk of bias. The predominant issues across all studies were the lack of allocation concealment (all 'U') and blinding (all 'N' for participants, therapists, and outcome assessors), which is common in behavioral intervention studies but introduces a risk of performance and detection bias.

Supplementary Material 5: Excluded High-Level Evidence (Guidelines, Systematic Reviews, and Expert Consensuses)

| No. | Source (Year) | Type | Exact Title | Reason for Exclusion |
| --- | --- | --- | --- | --- |
| 1 | NCCN (2024) | Guideline / Insights | NCCN Guidelines® Insights: Cervical Cancer, Version 1.2024 | P/O/S met, but I not met: Extensive guidance has been provided for cervical cancer radiotherapy, but specific recommendations for self-management of vaginal douching are lacking. |
| 2 | Chinese Society for Radiation Oncology (2025) | Guideline | Guidelines for Radical Radiotherapy of Cervical Cancer (2025 edition) | P/O/S met, but I not met: Focuses on radiotherapy techniques and complication prevention, without self-management irrigation protocols. |
| 3 | National Health Commission of China (2022) | Guideline | Guidelines for the Diagnosis and Treatment of Cervical Cancer (2022 edition) | P/O/S met, but I not met: Only broadly mentions nursing care, without specific content on vaginal irrigation self-management interventions. |
| 4 | WHO (2014 ) | Guideline | Comprehensive Cervical Cancer Control: A Guide to Essential Practice | P/O met, but I not met: Only mentions “gentle douching” in palliative care, without self-management protocols for radiotherapy patients. |
| 5 | ESGO–ESTRO–ESP (2023) | Guideline | ESGO–ESTRO–ESP Guidelines for the Management of Patients with Cervical Cancer | P/O/S met, but I not met: While vaginal douching is mentioned, specific guidance on its self-management is absent. |
| 6 | ASTRO (2020) | Guideline | Radiation Therapy for Cervical Cancer: Executive Summary of an ASTRO Clinical Practice Guideline | P/O met, but I not met: Focuses on dosage and technique, without behavioral self-management interventions. |
| 7 | BGCS (2021) | Guideline | British Gynaecological Cancer Society Cervical Cancer Guidelines: Recommendations for Practice | P/O/S met, but I not met:there is a lack of specific guidelines for self-management of vaginal douching. |
| 8 | ABS (2025) | Expert Consensus | The American Brachytherapy Society Consensus Guidelines on Brachytherapy for Cervical Cancer | P/O met, but I not met: there is a lack of specific guidelines for self-management of vaginal douching. |
| 9 | Chinese Expert Consensus (2020) | Expert Consensus | Chinese Expert Consensus on Image-Guided Three-Dimensional Brachytherapy for Cervical Cancer | P/O met, but I not met:there is a lack of specific guidelines for self-management of vaginal douching. |
| 10 | AIR | Position Paper | Prevention and Management of Radiotherapy-Related Toxicities in Gynecological Cancer Patients | P/O met, but I not met: Includes a review of vaginal toxicity management, but lacks RCT-level intervention evidence targeting irrigation adherence. |
